# Parents' views about healthcare professionals having real‐time remote access to their young child's diabetes data: Qualitative study

**DOI:** 10.1111/pedi.13363

**Published:** 2022-05-25

**Authors:** Barbara Kimbell, David Rankin, Ruth I. Hart, Janet M. Allen, Charlotte K. Boughton, Fiona Campbell, Elke Fröhlich‐Reiterer, Sabine E. Hofer, Thomas M. Kapellen, Birgit Rami‐Merhar, Ulrike Schierloh, Ajay Thankamony, Julia Ware, Roman Hovorka, Julia Lawton, KidsAP Consortium

**Affiliations:** ^1^ Usher Institute University of Edinburgh Edinburgh UK; ^2^ Wellcome Trust‐MRC Institute of Metabolic Science University of Cambridge Cambridge UK; ^3^ Department of Paediatrics University of Cambridge Cambridge UK; ^4^ Department of Paediatric Diabetes Leeds Children's Hospital Leeds UK; ^5^ Department of Paediatrics and Adolescent Medicine Medical University of Graz Graz Austria; ^6^ Department of Pediatrics I Medical University of Innsbruck Innsbruck Austria; ^7^ Hospital for Children and Adolescents University of Leipzig, Leipzig, Germany/Hospital for Children and Adolescents am Nicolausholz Bad Kösen Leipzig Germany; ^8^ Department of Pediatric and Adolescent Medicine, Comprehensive Center for Pediatrics Medical University of Vienna Vienna Austria; ^9^ Department of Pediatric Diabetes and Endocrinology Clinique Pédiatrique, Centre Hospitalier Luxembourg City Luxembourg; ^10^ Children's Services, Cambridge University Hospitals NHS Foundation Trust Addenbrooke's Hospital Cambridge UK

**Keywords:** children, data sharing, healthcare professionals, parents, qualitative, type 1 diabetes

## Abstract

**Objectives:**

We explored parents' views about healthcare professionals having remote access to their young child's insulin and glucose data during a clinical trial to inform use of data sharing in routine pediatric diabetes care.

**Research Design and Methods:**

Interviews with 33 parents of 30 children (aged 1–7 years) with type 1 diabetes participating in a randomized trial (KidsAP02) comparing hybrid closed‐loop system use with sensor‐augmented pump therapy. Data were analyzed using a qualitative descriptive approach.

**Results:**

Parents reported multiple benefits to healthcare professionals being able to remotely access their child's glucose and insulin data during the trial, despite some initial concerns regarding the insights offered into everyday family life. Key benefits included: less work uploading/sharing data; improved consultations; and, better clinical input and support from healthcare professionals between consultations. Parents noted how healthcare professionals' real‐time data access facilitated remote delivery of consultations during the COVID‐19 pandemic, and how these were more suitable for young children than face‐to‐face appointments. Parents endorsed use of real‐time data sharing in routine clinical care, subject to caveats regarding data access, security, and privacy. They also proposed that, if data sharing were used, consultations for closed‐loop system users in routine clinical care could be replaced with needs‐driven, ad‐hoc contact.

**Conclusions:**

Real‐time data sharing can offer clinical, logistical, and quality‐of‐life benefits and enhance opportunities for remote consultations, which may be more appropriate for young children. Wider rollout would require consideration of ethical and cybersecurity issues and, given the heightened intrusion on families' privacy, a non‐judgmental, collaborative approach by healthcare professionals.

## 1 INTRODUCTION

Type 1 diabetes management is particularly challenging in very young children due to rapid physical and social development, hypoglycemia unawareness, high insulin sensitivity, and unpredictable food intake and activity patterns.^1^, ^2^ Diabetes technologies, such as insulin pumps and continuous glucose monitors (CGMs), can help improve glycemic control and reduce severe hypoglycaemia^3^; hence, pediatric diabetes guidelines recommend their use.^4^ Use of diabetes technologies may also alleviate pressures on parents, with many reporting improved sleep, less worry about glucose excursions and hypoglycemia, more flexibility and freedom, and improved familial quality of life.^3^ As a result, technology use in this population is growing rapidly.^5^, ^6^

The latest CGM technology allows other individuals to access and remotely review users' glucose data, including healthcare professionals (HCPs). Little is known about users' experiences of, and views about, HCPs having remote access to their (or, in the case of parents, their child's) data. Studies suggest that data access may enhance communication by fostering a sense of intimacy and “seamless connectedness” between user and practitioner^7^ and, in turn, improve glycemic control.^8^ However, while access to glucose/insulin dose data can help HCPs provide better advice and support, the level of insight this allows into private routines and behaviors may make users feel scrutinized or judged.^9^, ^10^ Survey studies exploring remote data review and support by HCPs in pediatric populations have reported no notable impact on parent/child psychosocial outcomes, but suggest that it may substantially reduce HCP contact times during and between clinic visits.^11^, ^12^

Given the potential for the delivery of routine pediatric diabetes care to be enhanced by HCPs having remote data access, we sought to clarify the above equivocal findings. As part of a broader investigation of parents' experiences of using closed‐loop technology (as compared to sensor‐augmented pump therapy [SAP]) during a clinical trial (the KidsAP02 study), we explored their views about HCPs having remote access to their child's data during the trial. In doing so, we aimed to help inform use of data sharing with HCPs in routine pediatric diabetes care.

## 2 METHODS

We interviewed parents of young children aged 1–7 years with type 1 diabetes taking part in a 24‐month open‐label, multi‐center, multi‐national crossover trial (KidsAP02). Children were randomized to 16‐week use of a hybrid closed‐loop system (CLS) or 16‐week use of SAP therapy, before crossing over to the other regimen.^13^ In both arms, participants used a CGM sensor (Dexcom G6), which transmitted real‐time glucose levels to an unlocked personal smartphone hosting an app (CamAPS FX; CamDiab, Cambridge, UK) incorporating the closed‐loop control algorithm. Mealtime bolusing was initiated via the app, which in turn directed insulin delivery on the insulin pump (Dana Diabecare RS). Additionally, when closed‐loop mode was active, the control algorithm automatically adjusted insulin delivery rates via the insulin pump in response to real‐time sensor glucose levels.

In both arms, data from the app was automatically streamed to a cloud‐based platform (Glooko/Diasend; Göteborg, Sweden) every 5–10 min. These data were accessible to parents and study teams via both the Diasend app and the Diasend website, enabling near real‐time sharing of both insulin and glucose data with, and remote review by, HCPs working on the trial. As well as aiding trial evaluation, HCPs could use these data to inform clinical advice given to parents during pre‐arranged and unscheduled study contacts; for example, to optimize basal rates (during periods of SAP use) and/or mealtime ratios following transition onto the study equipment and during the trial. During each study period, HCPs telephoned/emailed parents twice during the first week and again after 1 week to: address any problems or concerns regarding the study devices, review device use, troubleshoot problems and provide additional training as necessary; then monthly, to troubleshoot problems and collect trial‐relevant information, for example, adverse events or device deficiencies. Parents could also contact HCPs via a 24‐h telephone helpline. Additionally, parents and participants attended two study appointments per study period: one at the start of each period for training purposes, and one at the end for collection of blood samples for HbA1c measurement. When the onset of the COVID‐19 pandemic necessitated a transition from face‐to‐face to remote modes of care delivery,^14^ increased emphasis was placed on using shared data to provide participants with support. See Table 1 for more information about the trial, devices used and remote data access arrangements.

**TABLE 1:** Information about the trial, devices used and remote data access arrangements

**The KidsAP02 trial** The KidsAP02 trial was conducted at seven clinical centers in four countries: Austria, Germany, Luxembourg, and the UK. Seventy‐four children (aged 1–7 years, type 1 diabetes duration ≥6 months, insulin pump use ≥3 months) were randomized to use either a hybrid closed‐loop system (intervention) or sensor‐augmented pump therapy (control) for 16 weeks. Following a “wash‐out” period (1–4 weeks), participants changed to the other regimen for a further 16 weeks. In both study arms, participants used the same component devices (pump, CGM sensor and smartphone). HCPs contacted parents 24 h after starting each study arm to address any immediate problems/concerns regarding the study devices, and again after a further 24–48 h and 1 week to review device use, troubleshoot problems and provide additional training as necessary. For the remainder of each study period, HCPs then telephoned/emailed parents monthly to troubleshoot problems and collect trial‐relevant information, such as adverse events or device deficiencies. Parents were also given access to a 24‐h telephone helpline for any problems related to the devices or general diabetes management. Additionally, parents and participants attended two study appointments per study period: one at the start of each period for training purposes, and one at the end for collection of blood samples for HbA1c measurement. **The CamAPS FX hybrid closed‐loop system** (CamDiab, Cambridge, UK) The CamAPS FX is a “hybrid” closed‐loop system, which combines automated, algorithm‐informed delivery of basal (background) insulin with user‐initiated mealtime boluses. CamAPS FX comprises the following devices/components: *DANA RS insulin pump* (Sooil, Seoul, South Korea), with CGM receiver; *Dexcom G6 factory‐calibrated real‐time CGM sensor* (Dexcom, San Diego, CA, USA), with CGM transmitter; *An unlocked Android smartphone (Galaxy S8, Samsung, South Korea)* running Android 8 OS or above, hosting the *CamAPS FX app* incorporating the Cambridge model predictive control algorithm (CamDiab, Cambridge, UK). The smartphone/app communicates wirelessly with both sensor and insulin pump, subject to being kept within 5–10 m of these devices. **Data‐sharing** The CamAPS FX app facilitates automatic data upload to a cloud‐based platform (Glooko/Diasend; Göteborg, Sweden), thus enabling data sharing and review by other individuals, including healthcare professionals, parents or other caregivers. Using participant login details, HCPs could view a child's real‐time data via the Diasend mobile app (requiring a smartphone) or the Diasend web application (requiring a computer). HCPs had remote access to the following shared data: “Real‐time” and retrospective graphs displaying glucose levels, rate of insulin delivery, meal‐time boluses and carbohydrate intake, high/low glucose range, Boost and Ease‐off status (for participants using the closed‐loop system), and a function which indicates whether the closed‐loop was operational (Automode on) or interrupted/switched off (Automode off). [Note: “Automode” remained switched off in the sensor‐augmented pump therapy arm of the trial; hence, during this phase, the hybrid closed‐loop system was not activated and rates/times of basal insulin delivery were instead pre‐set.] Summary statistics for daily, weekly, monthly or quarterly periods, including: average glucose, estimated HbA1c, time in/below/above target, number and average duration of hypos, total daily dose/bolus/basal insulin, and percentage of time in Automode (for those using the closed‐loop system). A summary clinic list of all study participants' data, including key glycemic metrics (time in range, time above and below target glucose range), insulin doses and system metrics, for example, closed‐loop use, CGM use and number of alarms.

The interview study was conducted by an independent team of qualitative researchers at the University of Edinburgh. Initially, the project researcher (B.K.; a female, PhD‐qualified, non‐clinical researcher) visited participating sites to gather contextual information and gain broad understanding and awareness of potential country/site‐specific differences. These insights helped inform the development of the interview topic guides and supported subsequent data analysis.

We used semi‐structured interviews informed by topic guides to help ensure the discussion remained relevant to the study aims, while offering participants flexibility to raise issues they considered important. The topic guides used open‐ended questions and probes and were informed by the site visits, literature reviews and input from clinical co‐investigators (via a dedicated qualitative working group comprising representation from all sites), and revised in light of emerging findings (see Table 2 for details of the main topic areas relevant to the reporting in this article). Data collection and analysis took place concurrently, allowing findings from earlier interviews to inform issues explored in subsequent ones.

**TABLE 2:** Relevant topic areas explored in the interviews

**Background information and pre‐trial diabetes management** Age of child with diabetes; general family set‐up (marital status, other children); parental employment Type of insulin pump and sensor used pre‐trial Parents' experiences of, and views about, diabetes care and support received pre‐trial **Professional support received during the trial** Experiences of, and views about, contact and support received from HCPs; did this contact/support differ when using CLS and if so, why; how might routine care be adapted for families using a CLS in everyday life Impact of COVID‐19 pandemic on seeking or receiving support during the trial: how did provision change during this time; what did they like/dislike about this and why; would they prefer more/less of this in future and why Parents' experiences of, and views about, HCPs having remote access to their child's real‐time data (e.g., concerns, perceived advantages/disadvantages) Experiences of, and views about, HCPs using remote data review to offer support Views and potential concerns about HCPs being given remote data access in routine diabetes care

### 2.1 Sampling and recruitment

Participants were recruited following randomization to the trial at seven clinical sites in four countries: Austria, Germany, Luxembourg, and the UK. Parents were consented into the interview study when they were consented into the trial. We employed purposive sampling to promote diversity with respect to participating countries and sites as well as the child's age and gender. Recruitment continued until data saturation was reached, that is, when new data ceased to generate new findings.

### 2.2 Data collection and analysis

Parents were interviewed at the end of the first study period (i.e., after 16 weeks of using CLS or SAP therapy), and at the end of the second study period (i.e., after 16 weeks of using the other regimen). We aimed to interview one parent per child; however, in the case of three children both parents wished to participate in joint interviews. Interviews were conducted by telephone in English or German by B.K. (who is fluent in both languages) between September 2019 and September 2020. Participants had no prior relationship with B.K. and were made aware of her status as an independent non‐clinical researcher; participants were also given firm reassurances of confidentiality to allow them to feel comfortable sharing negative experiences if relevant to them. Interviews averaged 70 minutes and were digitally recorded and transcribed for in‐depth analysis. Interviews conducted in German were translated and transcribed into English by professional translation services. To ensure accuracy, all transcripts were checked against the original interview audio files by B.K.

To reduce bias and enhance rigor, two team members (B.K. and J.L., a highly‐experienced non‐clinical qualitative researcher) independently analyzed the data using a qualitative descriptive approach, which seeks to provide rich, low‐inference descriptions of an event or experience.^15^, ^16^ This involved: (1) repeatedly reading and cross‐comparing transcripts and coding meaningful text units; (2) recording initial analytical reflections on the data; (3) discussing these interpretations and agreeing on the main areas of relevance; (4) further in‐depth analysis to develop more nuanced interpretations of the data. Two further qualitative team members (D.R. and R.I.H.), who were also familiar with the interview content, were available to mediate; however, discrepancies in data interpretation were minimal and resolved through discussion without need for third‐party arbitration. We used a qualitative analysis software package, NVivo11 (QSR International, Doncaster, Australia), to facilitate data coding and retrieval. Joint interviews were analyzed using the same approach as that used for single‐person interviews.

The trial, including this qualitative sub‐study, received approval from all relevant national regulatory authorities and ethics committees in the participating sites/countries. Our reporting is guided by the Standards for Reporting Qualitative Research (SRQR).^17^

## 3 RESULTS

We interviewed 33 parents of 30 children at the end of their first study period and 29 parents of 26 children at the end of the second. Four parents were lost to follow‐up: one could not be re‐contacted and three second interviews were not pursued due to staffing challenges caused by the COVID‐19 pandemic and consensus that data saturation had already been reached. Sample characteristics and details of the devices used pre‐trial are provided in Table 3.

**TABLE 3:** Sample characteristics and devices used by participants pre‐trial

Characteristic	*n*	%^a^	Mean (range)
**Parents** ^b^	**33**		
Mothers	25	75.8	
Fathers	8	24.2	
Married/co‐habiting	32	97.0	
Country of residence			
Austria	10	30.3	
Germany^c^	1	3.0	
Luxembourg	9	27.8	
United Kingdom	10	30.3	
Employment			
Full‐time	15	45.5	
Part‐time	13	39.4	
Full‐time carer	5	15.2	
Reduced hours/career break/quit employment due to diabetes care	9	27.3	
Occupation			
Professional	22	66.6	
Semi‐skilled	5	15.1	
Unskilled	1	3.0	
Full‐time carer	5	15.1	
**Children**	**30**		
Girls	13	43.3	
Boys	17	56.6	
Ethnicity			
White	28	93.3	
Mixed race	2	6.7	
Age at time of first interview; years			4.9 (2–8)
Age at time of diagnosis; years			2.2 (0.5–5)
Diabetes duration; years since diagnosis			2.7 (1–4.5)
Baseline HbA1c (%)			7.4 (6.1–9.0)
Living with siblings	24	80.0	
**Devices used before joining the trial**			
Insulin pumps:			
Medtronic MiniMed 640G	25	83.3	
Accu Chek	4	13.3	
Animas	1	3.3	
Sensors:			
Freestyle Libre flash monitor	2	6.7	
Medtronic Enlite/Guardian CGM	21	70.0	
Dexcom 4/5 CGM	2	6.7	
Dexcom 6 CGM	5	16.7	

^a^ Percentages may not add up to 100% due to rounding.

^b^ Of a total of 30 first‐round interviews, 22 were conducted with mothers, five with fathers and three were joint interviews with both parents. Of the 26 follow‐up interviews, 19 were conducted with mothers, four with fathers and three were joint interviews with both parents.

^c^ Only one parent could be recruited from Germany before recruitment into the interview study had to stop, due to the German site starting later on in the trial than other sites.

Below, we provide a brief description of children's diabetes care arrangements before the trial to set the context for understanding how, from parents' perspectives, these arrangements were enhanced by HCPs having remote access to their child's data during the trial. We then present parents' accounts of how data sharing benefitted themselves, their child and HCPs. Finally, we present parents' suggestions for how, in light of their trial experiences, data sharing could be used to enhance delivery of routine diabetes care for young children. As we found no differences in parents' experiences relating to their country of residence or the child's age or gender, we do not detail such characteristics in our reporting. Unless otherwise indicated, the parent quoted is a mother.

### 3.1 Pre‐trial care arrangements

Across all locations, parents reported similar pre‐trial routine diabetes care arrangements. These comprised regular (typically quarterly) face‐to‐face clinic appointments, where diabetes professionals recorded the child's height and weight, checked HbA1c levels, examined catheter and sensor sites, and reviewed glucose data to determine whether changes to basal rates or mealtime ratios were required. Additionally, parents described being able to contact their diabetes team between appointments (by telephone or email) if they had any questions or concerns, and how additional clinic appointments were made available to them if required:

“We have a clinic [appointment] every three months, basically, and then we have a clinic mobile phone number that we can call anytime from 8:30 in the morning until five, and then we have an out‐of‐hours number which we can call as well, 24 hours…They've given us extra clinic ones when we've needed to come and talk to them…So we feel very supported and it feels, basically, we can see people whenever we need to see people.” (027_CLS, father)

All parents praised these arrangements and described feeling well supported by their team, with whom many had long‐standing relationships by virtue of the child having been under their care since diagnosis.

### 3.2 Experiences of real‐time data sharing during the trial

HCPs delivering care during the trial were, for all but three families, the same individuals who provided the child's care in routine clinical practice. Despite this, some parents noted having “initially felt a bit funny” (030_CLS) about these individuals being able to access their child's insulin and glucose data, because these data gave insight into their routines and private life more generally. However, all of these parents notes how any initial discomfort about “being watched” had quickly abated:

“I think in the beginning I was a bit more concerned that they…can see, you know, especially the hours we were keeping, like: oh, they're going to see we were having breakfast late today. And he's going to bed late. But…as it went on I got used to it.” (011_SAP)

#### 3.2.1 Better understanding and more tailored advice

Parents also noted how these concerns had been substantially outweighed by the benefits gained by their child. Specifically, parents noted how, by virtue of being able to remotely access their child's data to inform pre‐scheduled and unplanned study contacts/visits, HCPs had had much better understanding and awareness of activity patterns, everyday life and insulin requirements than was possible in routine clinical care, when they had generally only reviewed “snapshots” (typically the last 1–2 weeks) of data at quarterly review appointments. It was also noted how, because of this enhanced awareness, staff could offer clinical input better tailored to their child's needs and circumstances.

“If you have an appointment with the doctor, they only look at what has happened one or two weeks beforehand, but it could be that there was something on, for example sick[ness] or I don't know‐ so this way they can look at it over a longer period of time. I think that's better.” (021_SAP)

#### 3.2.2 Lessened workload

Parents also highlighted multiple logistical benefits to remote data access/availability. Some, like this mother, welcomed no longer needing to remove the child's pump to access readings and email or upload these in advance of a consultation:

“This just makes it easier, because we're not having to sit for 20 minutes downloading on the computer, because it's automatically going to them…It was finding the time sometimes to download which was difficult, because he [child] obviously always has his pump on him, so it was during bath time, (father) would be bathing him and I'd be there trying to download everything as quickly as possible.” (022_SAP)

Others observed how HCPs no longer needed to spend time during consultations downloading directly from the child's pump or glucose meter. HCPs' ability to “simply push a button and…look how it has been” (017_CLS), as some parents noted, freed up valuable consultation time to review their child's data and determine necessary adjustments:

“Before…we had an appointment every three months and we just came, they downloaded the data from the pump, they had 10 minutes on checking the 3‐month record, let's say, and how can you adjust any improvement of set‐up in 10 minutes?” (015_SAP)

Parents also described how HCPs' ability to remotely access their child's data had simplified and facilitated the process of help‐seeking outside of pre‐arranged appointments, which, before the trial, had, for some, occurred “once to sometimes twice a week, because her levels were so hard to manage” (020_CLS). They observed finding it helpful that HCPs were able to instantly access the child's data on their own computers and “look at the same thing I'm looking at” (002_SAP), as this meant they no longer needed to remember, or gather and transfer, all the relevant details HCPs needed to formulate appropriate solutions:

“Before, if I would have called and been like: ‘I'm really struggling with her basal, I'd like some help.’…I have to verbalise it, I have to say what her levels have been at different times and everything. Or I have to get online and send them a document with the values and everything.” (025_SAP)

#### 3.2.3 Improved support

Most parents recounted how, during study contacts, especially those that had taken place in the initial days/weeks of using the study equipment, HCPs had alerted them to potential problems, such as a catheter being blocked, a mealtime ratio needing adjusted, or inappropriate device use. Parents reported welcoming these interventions, because “the more support one gets, the better” (019_CLS). Knowing that HCPs would regularly review their child's data during the trial was especially comforting to parents of recently‐diagnosed children, “cause we're still learning and we're still open to being taught how this all works” (004_SAP). Regular contact from HCPs was likewise welcomed by parents who professed to feeling reluctant to seek professional input for fear of being a burden, “because…sometimes, you know, we don't want to phone too much the team [sic], we know that they have a lot of patients” (016_SAP).

Parents also suggested that, when HCPs had offered input and support after having reviewed the child's data, this had been well received because of the sensitive and non‐judgmental approach they had employed, which was “never moralising, but more questioning why it was like that and should it be different in future” (010_CLS). Parents also described welcoming HCPs using a collaborative rather than prescriptive approach in these situations:

“They called a few times and were just kind of asking, like: ‘Okay, how's it going? We were looking at her blood sugars and we saw that you've been having this problem. How do you feel like it's been going that way?’ Like: ‘Should we be looking at that and changing it?’ and that. It's been a lot more [like] a conversation. It hasn't just been like they've called and been like: ‘I sent you an email with a new basal rate,’ or something like that.” (025_SAP)

#### 3.2.4 Reduced need for face‐to‐face consultations

Following the onset of the COVID‐19 pandemic, parents in all sites reported transitioning from face‐to‐face to remote (video or telephone) review appointments. There was widespread consensus that, because HCPs had been able to remotely access their child's data, the input given during these consultations had not been compromised:

“We had a telephone consultation instead of, obviously, a face‐to‐face, which was fine…we went through everything that we would have done in clinic. Obviously…he couldn't have his HbA1c checked, or his height and weight…but otherwise…we talked like we would have done face‐to‐face. So that was fine.” (008_CLS)

Furthermore, the majority of parents noted (un)anticipated benefits to this change. As well as not having to travel to the hospital (which could be time consuming, require some to take time off work and/or take the child out of school), parents noted quality‐of‐life benefits to their child by virtue of no longer having to attend disruptive, medicalized appointments where they were not active participants:

“With these phone calls…it's better for the patient, because she is less at hospital, so it's not that much in her face. And basically, at the age of five she is not taking part in it anyway, she sits in a corner. You know, she is measured and blood finger pricks and these things, but then she is basically sitting an hour and painting.” (017_CLS)

Several parents, like this father, also noted having gained more from remote consultations as it had been easier to keep the child entertained in their own home, and this had allowed them to concentrate and have more constructive conversations with HCPs:

“The questions that they ask or the questions that you ask…in the phone call and the Zoom call today, they have been answered. Whereas when you've got [child] there, you're kind of keeping half an eye on him and you're not able to give necessarily a full answer.” (022_CLS, father)

### 3.3 Potential adaptations to routine diabetes care for young children

The majority of parents endorsed continued use of data sharing in routine diabetes care, in part because it would allow more consultations to be undertaken remotely, which, following their pandemic experiences, parents now considered a more age‐appropriate approach. Parents also noted how, while using the closed‐loop system during the trial, they had needed much less clinical input to optimize their child's glycemic control, due to the system automatically regulating basal rates:

“It used to be‐ I would be ringing constantly about, saying: ‘Oh, his basal's sort of doing this, or his basal's doing that. Should we tweak it a bit? Should we do this?’ And you'd have a look and see what you think. And honestly, we had none of that.” (008_CLS)

Indeed, some suggested that, were more regular remote support to be made available to closed‐loop system users in routine care, they would favor a model of care whereby regular, scheduled review appointments could be replaced with ad‐hoc appointments as and when needed:

“‘Cause…I don't know what we're gonna talk about this afternoon [during review appointment], ’cause there's nothing to say. Like, everything's just going really, really well…So maybe if we just had it on a basis of: ‘Oh, we'll have a look, you know, we'll definitely have a look at her data every, you know, couple of months, and if we feel like there's a problem we'll call at that point,’ and we don't need like a particular scheduled appointment in.” (018_CLS)

As parents recognized that HCPs working in routine clinical care would have less time to check data than HCPs during the trial, some also endorsed use of specialist software alerting HCPs to potentially problematic events or patterns in the data:

“I would hope on a closed‐loop system there'd be a software that would be looking at what your closed‐loop system is reporting back to them, and then bring it to the attention of a human to have a look at.” (026_CLS, father)

#### 3.3.1 Concerns/caveats about HCPs having continuous data access

While most parents reported having no concerns regarding potential data protection or privacy breaches when granting HCPs continuous data access, several emphasized how their consent would be contingent on this being restricted to a select group of trusted individuals:

“…because I really trust those people there. I think they are really nice and we get on well. I think if you had a huge team there, it would be different.” (013_CLS, father)

Similarly, some parents described specific scenarios in which they would likely refuse HCP access to their child's data, for example, if the data were stored on an unsafe internet‐based platform or shared with third‐party organizations, or if HCPs were able to remotely intervene in insulin delivery. Others observed how, while they had few anxieties about granting data access while their child was still young, it would be important to take into consideration the child's own preferences as they grew older:

“I'm not sure…if he'll have the same idea if he was sort of a teenager and things like that, but I think at the age he is now…I think it's really helpful.” (008_CLS)

## 4 DISCUSSION

Parents saw HCPs' ability to remotely access their child's data during the trial as extending and enhancing pre‐trial care arrangements. Key benefits included: less work for themselves and HCPs uploading/downloading data, improved consultations and better support between consultations. Parents further noted how data sharing enabled diabetes consultations to be delivered remotely; these being seen as more suitable for young children than face‐to‐face consultations. Some parents also proposed that remote data access and review by HCPs could allow routine appointments for closed‐loop users to be replaced with needs‐driven, ad‐hoc consultations; this being due, in part, to their needing less support when using a closed‐loop system. In light of their positive experiences, parents recommended use of real‐time data sharing in routine clinical care, albeit subject to caveats related to data access, security and privacy.

Some parents reported initially feeling uneasy about HCPs having remote access to their child's data, due to the insights these data could offer into family routines and everyday life. Indeed, in this trial, HCPs were able access insulin as well as glucose data, which potentially provided richer insight into families' lives than CGM data alone. Given this invasion of privacy, some have argued that closed‐loop data sharing should be based on consent and agreement about the amount and type of data to be shared with HCPs.^9^ Additionally, ground rules could be agreed, and regularly reviewed, regarding if, when and how frequently HCPs will check these data.

In this study, data review was mostly undertaken by HCPs with whom parents had long‐standing relationships and, consistent with others' findings,^18^ many parents stressed how, in routine clinical care, they would want this input to be provided by their usual clinical team. However, making remote support contingent upon it being provided by known and trusted individuals (instead of, for example, a centralized specialist hub), may have logistical and cost implications. Reassuringly, however, in line with feedback from HCPs delivering closed‐loop system care,^19^, ^20^ parents reported a reduced need for clinical input when using a closed‐loop system and, consequently, combined with HCPs having remote data access, a preference for fewer routine appointments and more infrequent ad‐hoc support. Moreover, studies exploring the impact of HCPs providing regular (weekly or fortnightly) remote review and support to parents of older children and adults with diabetes have noted that this approach can be both cost‐effective^21^ and time‐efficient,^22^ and, by virtue of enabling pre‐emptive troubleshooting, reduce the time needed during clinic appointments.^11^ This suggests that providing regular remote data review/support might not necessarily add to HCPs' overall workload and could potentially reduce both the length and frequency of clinic visits; albeit this is an area where further research would be warranted. Alternatively, to lessen the impact on their workloads, HCPs could, as some parents proposed, utilize platforms that use algorithm‐based alarms to prompt data reviews at critical time points only, such as TreC Diabetes^7^; indeed, others have noted that the optimal frequency for providing remote input requires closer investigation.^12^ Finally, some commentators have warned that, despite its advantages, remote data review and support may not meet the needs of all populations.^23^ Our findings suggest that, in pediatric populations, remote data access may be particularly beneficial to parents of newly‐diagnosed and/or very young children, those feeling more anxious or reluctant to seek advice, and when the child first transitions onto new technologies. However, the benefits and widespread acceptability of this approach reported by all parents in this study indicate value in exploring how remote data review/support might be offered to all those caring for a child with type 1 diabetes who wish to use such provisioning.

Parents highlighted benefits to attending fewer face‐to‐face consultations, including fewer absences from school or work, which, as others have noted, can be disruptive to both parent and child and reduce productivity and learning.^24^ Given the short and long‐term health‐economic consequences of lost productivity and education, offering real‐time data sharing (which facilitates the remote delivery of consultations) in routine care may therefore also deliver wider societal benefits. Mirroring findings by Lawton et al.,^25^ parents in this study also noted how remote consultations helped lessen the distraction of keeping a young child entertained, thereby enabling more effective conversations with HCPs.

Wider rollout of remote diabetes support based on real‐time data sharing would require careful consideration and mitigation of several critical issues. For example, we recommend that HCPs offering input and support by remotely accessing real‐time glucose and insulin data employ a sensitive, non‐judgmental and collaborative conversational approach, as this was well received by parents in this study. Furthermore, to promote optimal uptake, technology developers and policymakers should consider potential ethical issues (e.g., balancing increased device complexity with user safety, or the availability of insurance/reimbursement options),^9^ and safeguard individuals' cybersecurity with regard to data theft and external device manipulation.^26^, ^27^ The parents in our study were mostly in professional occupations and, hence, were not in a state of digital poverty. However, it is important to recognize that individual and socioeconomic disparities exist regarding confidence with, and, importantly, access to, diabetes technologies, reliable internet connectivity and appropriate private space. Care providers therefore need to ensure that individuals most likely to benefit from remote care provision are supported to access this.^28^

A key study strength is the inclusion of parents of young children of different ages, living in different countries and subject to different healthcare systems. However, as individuals participating in a clinical trial are likely to be particularly motivated, their views may not necessarily reflect those of the wider population. A further potential limitation is that, in the context of a clinical trial, participants would have expected to have frequent contact with HCPs, which may not be practicable in clinical practice. Furthermore, reflecting a common limitation in clinical trials, our sample was heavily skewed towards white, married/co‐habiting parents in professional employment. Given the importance of ensuring fair and equitable access to diabetes technologies and patient‐centered modes of care delivery, future work should consider the perspectives of people from other socioeconomic and minority ethnic groups. As our findings are specific to parents of young children, we further recommend undertaking similar research with other diabetes populations (e.g., adults, adolescents, pregnant women), who may have different support needs and concerns about privacy; this will also help identify those most likely to benefit from such provisioning.^29^ Finally, research should explore HCPs' experiences of providing remote support and, importantly, their views about how such provisioning might be integrated into routine clinical care.

## 5 CONCLUSIONS

From parents' perspectives, real‐time data sharing can offer clinical, logistical and quality‐of‐life benefits and enhance opportunities for the delivery of remote consultations, which may be more suitable for young children. As well as consideration of ethical and cybersecurity issues, wider rollout would require HCPs to be sensitive to the heightened intrusiveness of continuous data access on family life and engage with parents in a non‐judgmental and collaborative manner.

## AUTHOR CONTRIBUTIONS

Julia Lawton conceived and designed this sub‐study. Barbara Kimbell collected the data, which were analyzed by Julia Lawton and Barbara Kimbell. Barbara Kimbell wrote the first draft of the manuscript. David Rankin, Ruth I. Hart, Janet M. Allen, Charlotte K. Boughton, Fiona Campbell, Elke Fröhlich‐Reiterer, Sabine E. Hofer, Thomas M. Kapellen, Birgit Rami‐Merhar, Ulrike Schierloh, Ajay Thankamony, Julia Ware, and Roman Hovorka reviewed, edited and approved the final version of the manuscript.

## CONFLICT OF INTEREST

R.H. reports having received speaker honoraria from Eli Lilly and Novo Nordisk, serving on advisory panels for Eli Lilly and Novo Nordisk, and receiving license fees from BBraun and Medtronic. R.H. also reports patents, patent applications, shareholding and directorship at CamDiab. C.K.B. has received consultancy fees from CamDiab. F.C. has attended advisory boards, obtained speaking fees and educational support from Abbott, Dexcom, Medtronic, Lilly, Insulet, and Novo Nordisk. E.F.R. reports having received speaker honoraria from Eli Lilly and Novo Nordisk, and serving on advisory boards for Eli Lilly and Sanofi. The authors B.K., D.R., R.I.H., J.M.A., S.E.H., T.M.K., B.R.M., U.S., A.T., J.W., and J.L. report no conflicts of interest relevant to this article.

## ETHICS STATEMENT

Approval for the trial and qualitative sub‐study was obtained from national regulatory authorities and relevant ethics committees in the participating sites/countries.

## Data Availability

Due to risks to participants’ privacy, the datasets (interview transcripts) supporting the findings of this study are not publicly available.

## References

[1] Streisand R , Monaghan M . Young children with type 1 diabetes: challenges, research, and future directions. Curr Diab Rep. 2014;14:520. 25009119 10.1007/s11892-014-0520-2 PMC4113115

[2] DiMeglio LA , Kanapka LG , DeSalvo DJ , et al. Time spent outside of target glucose range for young children with type 1 diabetes: a continuous glucose monitor study. Diabet Med. 2020;37(8):1308‐1315. doi:10.1111/dme.14276 32096282 PMC9065795

[3] Nevo‐Shenker M , Phillip M , Nimri R , Shalitin S . Type 1 diabetes mellitus management in young children: implementation of current technologies. Pediatr Res. 2020;87:624‐629. 31715623 10.1038/s41390-019-0665-4

[4] Sundberg F , Barnard K , Cato A , et al. ISPAD guidelines. Managing diabetes in preschool children. Pediatr Diabetes. 2017;18:499‐517. 28726299 10.1111/pedi.12554

[5] van den Boom L , Karges B , Auzanneau M , et al. Temporal trends and contemporary use of insulin pump therapy and glucose monitoring among children, adolescents, and adults with type 1 diabetes between 1995 and 2017. Diabetes Care. 2019;42:2050‐2056. 31488568 10.2337/dc19-0345

[6] Foster N , Beck R , Miller K , et al. State of type 1 diabetes management and outcomes from the T1D exchange in 2016‐2018. Diabetes Technol Ther. 2019;21:66‐72. 30657336 10.1089/dia.2018.0384 PMC7061293

[7] Piras EM , Miele F . On digital intimacy: redefining provider–patient relationships in remote monitoring. Sociol Health Illn. 2019;41:116‐131. doi:10.1111/1467-9566.12947 31599992

[8] Farmer AJ , Gibson OJ , Dudley C , et al. A randomized controlled trial of the effect of real‐time telemedicine support on glycemic control in young adults with type 1 diabetes (ISRCTN 46889446). Diabetes Care. 2005;28:2697‐2702. 16249542 10.2337/diacare.28.11.2697

[9] Quintal A , Messier V , Rabasa‐Lhoret R , Racine E . A critical review and analysis of ethical issues associated with the artificial pancreas. Diabetes Metab. 2019;45(1):1‐10. 29753624 10.1016/j.diabet.2018.04.003 PMC6202257

[10] Oikonomidi T , Ravaud P , James A , Cosson E , Montori V , Tran VT . An international, mixed‐methods study of the perceived intrusiveness of remote digital diabetes monitoring. Mayo Clin Proc. 2021;96(5):1236‐1247. doi:10.1016/j.mayocp.2020.07.040 33487438

[11] Gandrud L , Altan A , Buzinec P , et al. Intensive remote monitoring versus conventional care in type 1 diabetes: a randomized controlled trial. Pediatr Diabetes. 2018;9:1086‐1093. doi:10.1111/pedi.12654 29464831

[12] Marrero DG , Vandagriff JL , Kronz K , et al. Using telecommunication technology to manage children with diabetes: the Computer‐Linked Outpatient Clinic (CLOC) Study. Diabetes Educ. 1995;21(4):313‐319. doi:10.1177/014572179502100409 7621734

[13] Fuchs J , Allen JM , Boughton CK , et al. Assessing the efficacy, safety and utility of closed‐loop insulin delivery compared with sensor‐augmented pump therapy in very young children with type 1 diabetes (KidsAP02 study): an open‐label, multicentre, multinational, randomised cross‐over study protocol. BMJ Open. 2021;11:e042790. 10.1136/bmjopen-2020-042790 PMC7883854 33579766

[14] Sarteau AC , Souris KJ , Wang J , et al. Changes to care delivery at nine international pediatric diabetes clinics in response to the COVID‐19 global pandemic. Pediatr Diabetes. 2021;22:463‐468. doi:10.1111/pedi.13180 33470020 PMC8013674

[15] Sandelowski M . Whatever happened to qualitative description? Res Nurs Health. 2000;23:334‐340. 10940958 10.1002/1098-240x(200008)23:4<334::aid-nur9>3.0.co;2-g

[16] Sandelowski M . What's in a name? Qualitative description revisited. Res Nurs Health. 2010;33:77‐84. 20014004 10.1002/nur.20362

[17] O'Brien BC , Harris IB , Beckman TJ , et al. Standards for reporting qualitative research: a synthesis of recommendations. Acad Med. 2014;89(9):1245‐1251. 24979285 10.1097/ACM.0000000000000388

[18] von Sengbusch S , Doerdelmann J , Lemke S , et al. Parental expectations before and after 12‐month experience with video consultations combined with regular outpatient care for children with type 1 diabetes: a qualitative study. Diabet Med. 2021;38:e14410. doi:10.1111/dme.14410 32969088

[19] Kimbell B , Rankin D , Ashcroft NL , et al. What training, support and resourcing do health professionals need to support people using a closed‐loop system? A qualitative interview study with health professionals involved in the closed loop from onset in type 1 diabetes (CLOuD) trial. Diabetes Technol Ther. 2020;22(6):468‐475. doi:10.1089/dia.2019.0466 32048877 PMC7262645

[20] Farrington C , Murphy HR , Hovorka R . A qualitative study of clinician attitudes towards closed‐loop systems in mainstream diabetes care in England. Diabet Med. 2020;37(6):1023‐1029. 31943318 10.1111/dme.14235 PMC7317734

[21] Chase HP , Pearson JA , Wightman C , Roberts MD , Oderberg AD , Garg SK . Modem transmission of glucose values reduces the costs and need for clinic visits. Diabetes Care. 2003;26(5):1475‐1479. 12716807 10.2337/diacare.26.5.1475

[22] Cho J‐H , Chang S‐A , Kwon H‐S , et al. Long‐term effect of the internet‐based glucose monitoring system on HbA1c reduction and glucose stability. Diabetes Care. 2006;29(12):2625‐2631. doi:10.2337/dc05-2371 17130195

[23] Walker RC , Tong A , Howard K , Palmer SC . Patient expectations and experiences of remote monitoring for chronic diseases: systematic review and thematic synthesis of qualitative studies. Int J Med Inform. 2019;124:78‐85. doi:10.1016/j.ijmedinf.2019.01.013 30784430

[24] Katz ML , Laffel LM , Perrin JM , Kuhlthau K . Impact of type 1 diabetes mellitus on the family is reduced with the medical home, care coordination, and family‐centered care. J Pediatr. 2012;160(5):861‐867. 22133424 10.1016/j.jpeds.2011.10.010 PMC3328639

[25] Lawton J , Waugh N , Noyes K , et al. Improving communication and recall of information in paediatric diabetes consultations: a qualitative study of parents' experiences and views. BMC Pediatr. 2015;15:67. doi:10.1186/s12887-015-0388-6 26054649 PMC4460975

[26] Thiel S , Mitchell J , Williams J . Coordination or collision? The intersection of diabetes care, cybersecurity, and cloud‐based computing. J Diabetes Sci Technol. 2017;11(2):195‐197. doi:10.1177/1932296816676189 27784829 PMC5478031

[27] O'Keeffe DT , Maraka S , Basu A , Keith‐Hynes P , Kudva YC . Cybersecurity in artificial pancreas experiments. Diabetes Technol Ther. 2015;17(9):664‐666. doi:10.1089/dia.2014.0328 25923544 PMC4556085

[28] Monaghan M , Marks B . Personal experiences with COVID‐19 and diabetes technology: all for technology yet not technology for all. J Diabetes Sci Technol. 2020;14(4):762‐763. doi:10.1177/1932296820930005 32460543 PMC7673164

[29] Mecklai K , Smith N , Stern AD , Kramer DB . Remote patient monitoring ‐ overdue or overused? N Engl J Med. 2021;384:1384‐1386. doi:10.1056/NEJMp2033275 33853209

